# Low Cardiorespiratory Fitness Post-COVID-19: A Narrative Review

**DOI:** 10.1007/s40279-022-01751-7

**Published:** 2022-09-17

**Authors:** Fabian Schwendinger, Raphael Knaier, Thomas Radtke, Arno Schmidt-Trucksäss

**Affiliations:** 1 Division of Sports and Exercise Medicine, Department of Sport, Exercise and Health, University of Basel, Grosse Allee 6, 4052 Basel, Switzerland; 2 Division of Sleep Medicine, Harvard Medical School, Boston, MA USA; 3 Medical Chronobiology Program, Division of Sleep and Circadian Disorders, Departments of Medicine and Neurology, Brigham and Women’s Hospital, Boston, MA USA; 4 Epidemiology, Biostatistics and Prevention Institute (EBPI), University of Zurich, Zurich, Switzerland

## Abstract

Patients recovering from COVID-19 often report symptoms of exhaustion, fatigue and dyspnoea and present with exercise intolerance persisting for months post-infection. Numerous studies investigated these sequelae and their possible underlying mechanisms using cardiopulmonary exercise testing. We aimed to provide an in-depth discussion as well as an overview of the contribution of selected organ systems to exercise intolerance based on the Wasserman gears. The gears represent the pulmonary system, cardiovascular system, and periphery/musculature and mitochondria. Thirty-two studies that examined adult patients post-COVID-19 via cardiopulmonary exercise testing were included. In 22 of 26 studies reporting cardiorespiratory fitness (herein defined as peak oxygen uptake—*V*O_2peak_), *V*O_2peak_ was < 90% of predicted value in patients. *V*O_2peak_ was notably below normal even in the long-term. Given the available evidence, the contribution of respiratory function to low *V*O_2peak_ seems to be only minor except for lung diffusion capacity. The prevalence of low lung diffusion capacity was high in the included studies. The cardiovascular system might contribute to low *V*O_2peak_ via subnormal cardiac output due to chronotropic incompetence and reduced stroke volume, especially in the first months post-infection. Chronotropic incompetence was similarly present in the moderate- and long-term follow-up. However, contrary findings exist. Peripheral factors such as muscle mass, strength and perfusion, mitochondrial function, or arteriovenous oxygen difference may also contribute to low *V*O_2peak_. More data are required, however. The findings of this review do not support deconditioning as the primary mechanism of low *V*O_2peak_ post-COVID-19. Post-COVID-19 sequelae are multifaceted and require individual diagnosis and treatment.

**Supplementary Information:**

The online version contains supplementary material available at 10.1007/s40279-022-01751-7.

## Key Points

**Table:** 

Exercise intolerance post-COVID-19 may likely have several causes and is not solely explained by deconditioning.
Peripheral followed by cardiovascular factors as well as lung diffusion limitations are central for long-term sequelae.
This work will improve the understanding of possible underlying mechanisms of low cardiorespiratory fitness post-COVID-19 and at the same time promote cardiopulmonary exercise testing as a valuable diagnostic tool in patients post-COVID-19. Based on this, more targeted rehabilitation programmes could be developed in the future.

## Introduction

Coronavirus disease 2019 (COVID-19) constitutes a tremendous burden for health care systems worldwide. Despite great research efforts and state-of-the-art treatment, approximately 15% of patients post-COVID-19 present with physiological and psychological symptoms of exhaustion (i.e. dyspnoea, fatigue, dizziness) persisting for several months post-infection [1]. These sequelae may be less frequent in individuals that received at least two doses of a two-dose COVID-19 vaccine regimen [2]. Cardiorespiratory fitness (CRF, herein defined as peak oxygen uptake—$\dot{V}$O_2peak_), a powerful predictor of mortality and risk factor for the development of numerous diseases [3], is commonly below normal in these patients, even up to 9 months post-infection [4–6]. Simultaneously, $\dot{V}$O_2peak_ is inversely associated with the risk of severe COVID-19 and hospitalisation [7].

**Fig. 1:**
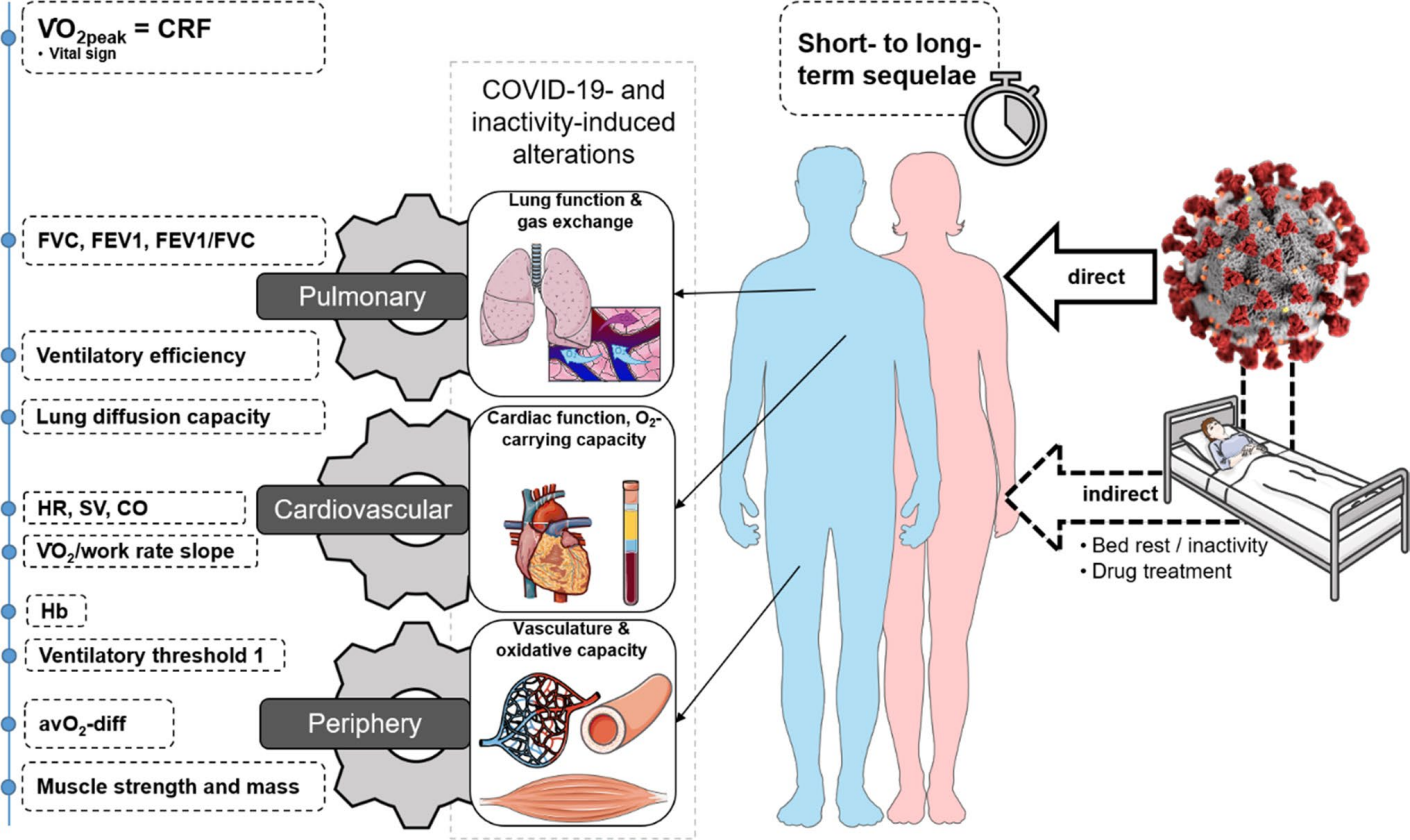
The figure illustrates leverage points through which COVID-19 could directly and/or indirectly induce low $\dot{V}$O_2peak_ and exercise intolerance in patients post-COVID-19. Parameters on the left side of the figure reflect the status quo of the gears (i.e. pulmonary system, cardiovascular system and periphery) and are indicative of organ limitations. The original concept of the gear system explaining determinants of $\dot{V}$O_2peak_ is available in Wasserman [9]. *a-vO*_*2*_* diff* arteriovenous oxygen difference. *CO* cardiac output, *COVID-19* coronavirus disease 2019, *CRF* cardiorespiratory fitness, *FEV1* forced expiratory volume in 1 s, *FVC* forced vital capacity, *Hb* haemoglobin, *HR* heart rate, *SV* stroke volume, $\dot{V}$*O*_*2peak*_ peak oxygen uptake

$\dot{V}$O_2peak_ represents the maximal oxidative metabolic capacity or maximal oxygen supply dependent on the aerobic fitness and health status of an individual [8, 9]. $\dot{V}$O_2peak_ relies on the interplay between the three gears—pulmonary system, cardiovascular system, and periphery/skeletal musculature system and mitochondria [8, 9]. The *Gear system* was originally introduced by Wasserman describing the interdependency of these three gears that together determine $\dot{V}$O_2peak_. It also helps to explain the origin of impaired $\dot{V}$O_2peak_ (see Fig. 1).

Recent studies have focused on uncovering potential underlying mechanisms of short- to long-term consequences of COVID-19 for $\dot{V}$O_2peak_. However, to the best of our knowledge, there is no review providing an in-depth overview for researchers, clinicians, and healthcare professionals.

Thus, this narrative review aims to fill this research gap. Furthermore, the aim was to discuss the contribution of the three gears as well as their alterations to low $\dot{V}$O_2peak_.

## Methods

A narrative review with a systematic approach was chosen as the study design because the aim was to primarily improve the understanding of COVID-19-related sequelae for $\dot{V}$O_2peak_ and possible underlying mechanisms, while secondarily constituting an exhaustive summary of pertinent literature. In systematic reviews, the focus is usually on the latter.

The databases Medline, EMBASE (on Ovid) and Covid-19 L^.^OVE by Epistemonikos were searched using the search strategies presented in Online Resource 1 (see electronic supplementary material [ESM]) through January 2022 to identify key publications. Furthermore, backwards and forwards citation tracking was done for all included studies. Relevant articles had to assess $\dot{V}$O_2peak_ by cardiopulmonary exercise testing (CPET) in adults. There were no language restrictions. Case studies/series and conference abstracts were excluded. For this review, we defined short term as up to 1 month, moderate term as > 1 to 5 months, and long term as > 5 months after COVID-19 diagnosis or hospital discharge [10].

The searches yielded a total of 139 studies. After deduplication in EndNote X9.3.3 (Clarivate, Philadelphia, USA), 106 studies were screened for eligibility. The Rayyan web application [11] was used to document the decision of inclusion or exclusion. Twenty studies fulfilled the inclusion criteria. Citation tracking produced 12 additional studies. After applying the eligibility criteria, we ultimately included 32 studies in this review. Of those, 29 studies included patients hospitalised due to COVID-19 (at least moderate COVID-19) [12]. Three studies examined elite athletes. These are presented in Table 1 but not discussed explicitly as they are not representative of the general population (i.e. considerably higher $\dot{V}$O_2peak_, better health status, lower median age) [13–15]. The total number of patients with CPET data was 1817. Four studies provided longitudinal data [13, 16–18].

**Table 1:** Characteristics, reported outcomes and authors’ conclusions of studies examining the short-term sequelae of COVID-19 (sorted in descending order by time since hospital discharge/infection)

Study	Patients post-COVID-19 (*n*) Median (SD) age (y), male sex (%) Median (SD) hospital stay (d)	COVID-19 severity	Inclusion/exclusion criteria	Outcomes	Authors’ conclusion: reason for low CRF
Csulak et al. [13]	*N* = 14 elite swimmers with mild COVID-19 23 ± 3 y, 50% male NR ex-/smoker = NR diabetes = NR Controls: *N* = 32 elite swimmers without symptoms and positive RT-PCR or antibody test	Mild	Inclusion: positive RT-PCR or antibody test for SARS-CoV-2; membership in professional teams taking part in national and international competition	Assessed at post-quarantine: $\dot{V}$O_2peak_ ⇔ $\dot{V}$E/$\dot{V}$CO_2_ ⇔ HR_max_ ⇔ O_2_ pulse ⇔ (Hb NR)	COVID infection with short-term detraining did not affect the performance of well-trained swimmers
Cavigli et al. [15]	*N* = 90 competitive athletes with asymptomatic or mild COVID-19 24 ± 10 y, 71% male NR ex-/smoker = NR diabetes = NR	Mild	Inclusion: asymptomatic of mild COVID-19 Exclusion: athletes with severe infection requiring hospitalisation or veteran athletes (i.e. > 50 years of age)	Assessed directly after acute COVID-19: $\dot{V}$O_2peak_ ⇔ ⇓ (95% of pred.) Spirometry parameters ⇔ Breathing reserve ⇔ $\dot{V}$E/$\dot{V}$CO_2_ ⇔ VT1 ⇔ (53% of $\dot{V}$O_2peak_) HR_max_ ⇓ (90% of pred.) O_2_ pulse ⇔ (Hb ⇔)	No cardiopulmonary limitations detected. Cardiac consequences of SARS-CoV-2 infection were found in 3.3% of competitive athletes
Anastasio et al. [14]	*N* = 13 elite cross-country skiers 21 ± 5 y, 77% male NR ex-/smoker = 0%/0% diabetes = NR Controls: *N* = 13 age, sex and days of inactivity-matched elite cross-country skiers without COVID-19-related symptoms and evidence of SARS-CoV-2 infection	Mild	Inclusion: positive RT-PCR test for SARS-CoV-2; membership in professional teams taking part in national and international competition Exclusion: dyspnoea, shortness of breath at rest or during mild exercise, peripheral oxygen saturation ≤ 95%, clinical or radiographic evidence of lower respiratory tract disease, respiratory rate > 30 breaths per minute, acute infection, hospitalisation due to COVID-19, treatment with steroid or antiviral agents	Assessed directly after acute COVID-19: $\dot{V}$O_2peak_ ⇔ (57.3 vs 56.9 mL^.^min^−1.^kg^−1^) Spirometry parameters ⇔ Breathing reserve ⇔ HR_max_ ⇓ (184 vs 196 bpm^#^) O_2_ pulse ⇔ (Hb NR) VT1 ⇓ (50% of $\dot{V}$O_2peak_#)	An early switch to anaerobic metabolism combined with unaltered cardiac and respiratory parameters might suggest a peripheral (muscular) aetiology of low aerobic performance (early VT1)
Baratto et al. [24]	*N* = 18 66 (21) y, 72% male 30 (10) days ex-/smoker = NR/17% diabetes = 6% Controls: *N* = 18, age-, sex- and body mass index-matched	Severe (*n* = 13), critical (*n* = 5)	Inclusion: judged clinically healed and weaned from oxygen, COVID-19 pneumonia Exclusion: pre-existing cardiac, respiratory, or musculoskeletal comorbidities, or cognitive decline; unable to perform CPET	Assessed at hospital discharge: $\dot{V}$O_2peak_ ⇓# (59% of pred.; 30% lower than controls) Spirometry parameters ⇓#, $\dot{V}$E/$\dot{V}$CO_2_ slope ⇔ ⇑# (mean slope: 32) $\dot{V}$O_2_/WR slope ⇓# (8.1 vs 10.9) HR_peak_ ⇓# O_2_ pulse ⇓# Cardiac output ⇔ ⇑ a-vO_2_ diff ⇓#	Mainly peripheral factors (anaemia and peripheral oxygen extraction)
Blokland et al. [40]	*N* = 23 59 (NR) y, 83% male 31 (NR) days ex-/smoker = NR/NR diabetes = NR	Moderate (*n* = 1), severe to critical (*n* = 22)	Inclusion: medically stable and expected to be discharged Exclusion: ongoing cardiac monitoring or ventilation; contraindications for CPET	Assessed at hospital discharge: $\dot{V}$O_2peak_ ⇓ (57% of pred.) Spirometry parameters ⇓ HR_peak_ ⇔ (93% of pred.) O_2_ pulse ⇓ (65% of pred.)	Limitations of CRF: ventilatory (*n* = 7), peripheral (*n* = 16) (i.e. reduced muscle mass)
Gao et al. [54]	*N* = 10 51 ± 17 y, 70% male NR ex-/smoker = NR/NR diabetes = NR	Moderate (*n* = 3), severe (*n* = 2), critical (*n* = 5)	NR	Assessed 30 days after discharge: $\dot{V}$O_2peak_ ⇓ (66% of pred.) Spirometry parameters ⇔ DLCO impaired in 3/10 cases (82% of pred.) VT1 ⇔ (47% of pred. $\dot{V}$O_2peak_) O_2_ pulse ⇓ (78% of pred.) (Hb NA)	Cardiac dysfunction, respiratory impairment, gas transfer inefficiency, and extra pulmonary factors were ruled out
Kersten et al. [41]	*N* = 231 48 ± 15 y, 43% male NR ex-/smoker = 20% diabetes = 6% CPET sample (*N* = 36): asymptomatic or mild (*n* = 31), moderate to critical (*n* = 5)	Asymptomatic (*n* = 34), mild (*n* = 179), moderate to critical (*n* = 18)	Inclusion: persistent symptoms Exclusion subsample: unacceptable symptom burden	Assessed minimum 30 days after discharge: Spirometry parameters ⇔ (< 80% of pred. in 10%) DLCO ⇔ (< 80% of pred. in 20%) CPET-subsample: No limitations in 45% Deconditioning in 13% Cardiac limitations in 3% Pulmonary-mechanical (16%) Pulmonary vascular (19%)	No specific conclusions regarding potential underlying mechanisms of low CRF

COVID-19 severity was categorised according to the World Health Organisation interim guidance [12] whenever possible. Colour coding: Dots from left to right represent mild (not hospitalised), moderate, severe and critical COVID-19, respectively. If a dot is coloured, this group is included in the particular study

*a-vO*_*2*_* diff* arteriovenous oxygen difference, *bpm* beats per minute, *COVID-19* coronavirus disease 2019, *CPET* cardiopulmonary exercise testing, *CRF* cardiorespiratory fitness, *DLCO* lung diffusion capacity using carbon monoxide, *Hb* haemoglobin, *HRR* heart rate reserve, *HR*_*max*_ maximal heart rate, *HR*_*peak*_ peak heart rate, *NR* not reported, *pred.* predicted, *SD* standard deviation, $\dot{V}$*E/*$\dot{V}$*CO*_*2*_ ventilatory efficiency, $\dot{V}$*O*_*2peak*_ peak oxygen uptake, *VT1* ventilatory threshold 1 (defined in [16]), *WR* work rate, # significantly different from control group; ⇑ increased; ⇓ decreased; ⇔ normal, ⇔ ⇑ slightly increased; ⇔ ⇓ slightly decreased

## Results and Discussion

### Cardiorespiratory Fitness

**Fig. 2:**
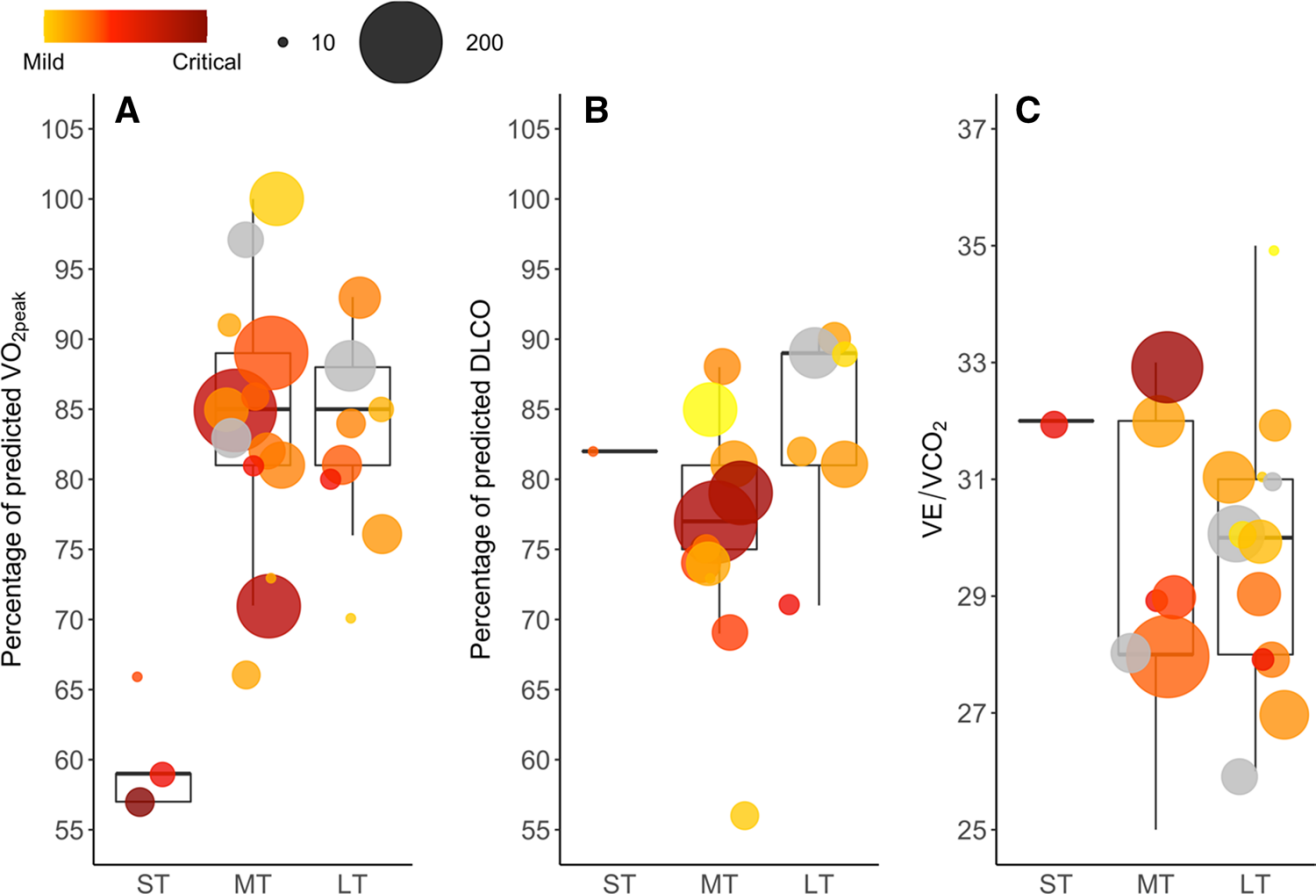
Medians of $\dot{V}$O_2peak_ (**A**), DLCO (**B**) and V̇E/V̇CO_2_ (**C**) weighted by sample size of respective studies or study visits in case a cohort was tested several times. Bubble area represents the sample size of studies. Bubble colour reflects COVID-19 severity of the majority of patients in the respective study (yellow = mild, dark red = critical). Grey bubbles: classification according to COVID-19 severity not possible. *DLCO* lung diffusion capacity using carbon monoxide, *LT* long term, *MT* medium term, *ST* short term, *V̇E/V̇CO*_*2*_ ventilatory efficiency, $\dot{V}$*O*_*2peak*_ peak oxygen uptake

In 22 out of 26 studies (two studies are counted twice as they reported a second measurement point) examining patients post-COVID-19, $\dot{V}$O_2peak_ of patients was < 90% of predicted. Figure 2A illustrates that $\dot{V}$O_2peak_ is markedly low shortly after hospital discharge. Furthermore, it is remarkable that even in the long term $\dot{V}$O_2peak_ is noticeably low. Interestingly, there seems to be no change in $\dot{V}$O_2peak_ from mid- to long-term follow-up, which could be due to the following reasons: (i) inactivity-related effects during the hospital stay [19, 20], (ii) direct COVID-19-related sequelae [21, 22], (iii) side effects of drug treatment [23, 24], and (iv) pre-morbid $\dot{V}$O_2peak_ may have been low. Regardless of whether $\dot{V}$O_2peak_ is low due to a past COVID-19 infection or if patients with lower $\dot{V}$O_2peak_ are more likely to have more severe disease progression, improving $\dot{V}$O_2peak_ will most likely reduce the risk of all-cause mortality as well as the risk for non-communicable diseases in these patients in the future [3]. The following sections will discuss the contribution of the three gears to low $\dot{V}$O_2peak_.

### Pulmonary System (Gear 1)

The pulmonary system is a central component in the supply chain of oxygen delivery to the working muscles [9]. While the pulmonary system usually is not the limiting factor of $\dot{V}$O_2peak_ in healthy individuals at sea level [8], it may well be in certain patient populations such as chronic obstructive pulmonary disease patients [25]. Ventilatory disorders are traditionally diagnosed using spirometry at rest measuring forced expiratory volume in 1 s (FEV_1_), forced vital capacity (FVC) and the ratio of the two (FEV_1_/FVC) [26]. Using CPET, ventilatory and pulmonary-vascular limitations are recognised by a reduced ventilatory capacity (breathing reserve at peak exercise), an abnormal ventilatory response, ventilatory inefficiency, or ventilation/perfusion mismatching (panels 1, 4, 6, 7, 9 in the original Wasserman 9-panel graph) [27].

A recent meta-analysis included 894 patients recovering from COVID-19 and presenting with abnormalities in lung function [28]. The prevalences of low total lung capacity, low FVC and low FEV_1_ were 14%, 12% and 7%, respectively [28]. These findings are in line with the studies summarised in Tables 1, 2, 3 that reported low $\dot{V}$O_2peak_ [4, 5, 17, 29–39]. On the day of hospital discharge, impaired lung function has been reported in only two studies [24, 40]. Overall, within the first months post-infection, restrictive lung disease does not seem to be responsible for low $\dot{V}$O_2peak_ [5, 18, 29–31, 41–44]. A German cohort study performed body plethysmography in 443 mainly non-hospitalised patients at a median of 10 months post-infection as well as in healthy controls [45]. Patients showed somewhat lower total lung capacity (99.1% vs 102.4% of predicted; *p* = 0.014) and higher airway resistance (77.3% vs 69.8% of predicted; *p* = 0.001) than controls [45]. Lung capacity, in contrast to airway resistance, however, is unlikely to affect $\dot{V}$O_2peak_ due to the overcapacity of human lungs [8, 46]. In addition, impairments in lung function cannot be ruled out by solely comparing them to normative data. Thus, pre-infection data are needed.

**Table 2:** Characteristics, reported outcomes, and author’s conclusions of studies examining the moderate-term sequelae of COVID-19 (sorted in descending order by time since hospital discharge/infection)

Study	Subjects (*n*) Median (SD) age (y), male sex (%) Median (SD) hospital stay (d)	COVID-19 severity	Inclusion/exclusion criteria	Outcomes	Authors’ conclusion: reason for low CRF
Raman et al. [42]	*N* = 58 55 ± 13 y, 59% male 9 (12) days ex-/smoker = 35% diabetes = 15% Controls: *N* = 30, matched for age, sex, body mass index, risk factors (i.e. smoking, hypertension, diabetes, coronary artery disease, and stroke)	Moderate (*n* = 37), severe (*n* = 21)	Inclusion: moderate to severe RT-PCR-confirmed COVID-19; enrolment did not rely on the presence of multi-organ symptoms; hospitalised individuals with moderate to severe COVID-19 Exclusion: contraindications to magnetic resonance imaging, severe comorbidities (i.e. end-stage renal, cardiac, liver, neurological disease	Assessed at 1.6 months post-disease onset: $\dot{V}$O_2peak_ ⇓# (81% of pred.) Spirometry parameters ⇔# DLCO ⇔⇓ (mean: 33.4; 81% of pred.) (impaired in 52%) $\dot{V}$E/$\dot{V}$CO_2_ slope ⇔ ⇑ # (mean slope: 32) O_2_ pulse ⇔ (Hb NR)# Left and right ventricular function not different between patients and controls, MRI markers elevated compared with controls (myocardial injury in 1/3 of patients) VT1 ⇔ ⇓ (41% of $\dot{V}$O_2max_ pred.#; 42% below 40% of $\dot{V}$O_2max_ pred.)	Low CRF was associated with blood inflammatory markers. Deconditioning was prominent among patients. Myocardial injury in one-third of patients. Muscle wasting secondary to catabolic state induced by severe illness may contribute to low CRF
Everaerts et al. [18]	*N* = 22 undergoing outpatient rehab: 55 (14) y 29 (27) days ex-/smoker = NR/NR diabetes = 18%	Moderate (*n* = 7) and severe to critical (*n* = 15)	Inclusion: referred by treating physician at discharge, on occasion of follow-up visit 6 weeks post-discharge by GP, or after inpatient rehabilitation; limb muscle force or 6-min walking distance < 70% of pred.; provided symptoms and functional status had deteriorated by COVID-19 Exclusion: elderly patients with severe functional or cognitive impairment, or patients in need of inpatient rehabilitation	Assessed at median 47 days post-discharge: $\dot{V}$O_2peak_ ⇓ (66% of pred.) Spirometry parameters ⇔ ⇓ DLCO ⇓ (56% of pred.) 3 months post-baseline (*n* = 16): $\dot{V}$O_2peak_ ⇔ ⇓ (91% of pred.) Spirometry parameters ⇔ DLCO ⇓ (75% of pred.)	No specific conclusions regarding potential underlying mechanisms of low CRF
Motiejunaite et al. [43]	*N* = 114 57 (18) y, 77% male 10 (12) days ex-/smoker = 27%/3% diabetes = 22%	Hospitalised (*n* = 104), mechanical ventilation (*n* = 21) corresponds to moderate, severe, and critical—further categorisation not possible	Inclusion: initial diagnosis of COVID-19 Exclusion: NR	Assessed at 3 months post-diagnosis: $\dot{V}$O_2peak_ ⇓ (71% of pred.) Spirometry parameters ⇔ ⇓ DLCO ⇓ (79% of pred.) $\dot{V}$E/$\dot{V}$CO_2_ slope ⇔⇑ (mean slope: 33; elevated in 32% of patients) $\dot{V}$O_2_/WR slope ⇔ ⇓ (9.3) Normal cardiac function O_2_ pulse ⇓ (79% of pred.) (Hb NR)	Deconditioning as the main mechanism. However, exercise hyperventilation should not be overlooked while exploring the causes of dyspnoea
Clavario et al. [29]	*N* = 200 59 (14) y, 57% male 17 (NR) days ex-/smoker = NR/43% diabetes = 6%	Moderate to severe—further categorisation not possible	Inclusion: admitted to COVID-19 wards with RT-PCR-confirmed infection Exclusion: NR	Assessed at 3 months post-discharge: $\dot{V}$O_2peak_ ⇓ (85% of pred.) Spirometry parameters ⇔ DLCO ⇓ (77% of pred.) HR_peak_ ⇔ ⇓ (92% of pred.) VT1 ⇔ (68% of $\dot{V}$O_2peak_)	Deconditioning (periphery) in one-third of patients likely linked to lower a-vO_2_ diff followed by cardiac limitations in 34%
Skjørten et al. [5]	*N* = 156 56 ± 13 y, 61% male 6 (8) days ex-/smoker = 40%/1% diabetes = 9%	Severe to critical (*n* = 31), 65% intubated and mechanically ventilated, moderate (*n* = 125)	Inclusion: age ≥ 18 years; admitted for > 8 h with a discharge diagnosis (International Statistical Classification of Diseases and Related Health Problems 10) of U07.1 (COVID-19, virus identified), U07.2 (COVID-19, virus unidentified) or J12.x (viral pneumonia, in combination with positive SARS-CoV-2 identification in nasopharyngeal swab) before 1 June 2020 Exclusion: prior diagnosis of chronic obstructive pulmonary disease, myocardial infarction, heart failure or peripheral arterial disease; living outside the hospitals’ catchment areas; inability to provide informed consent; participating in the World Health Organization Solidarity trial	Assessed at 3 months post-discharge: $\dot{V}$O_2peak_ ⇓ (89% of pred.) Spirometry parameters ⇔ ⇓ DLCO ⇔ ⇓ (84% of pred.) $\dot{V}$E/$\dot{V}$CO_2_ slope ⇔ # (mean slope: 28; elevated in 15% of patients) Absolute HR_max_ ⇓ HR_peak_ as % of pred. ⇔ O_2_ pulse ⇔ (Hb NR) VT1 ⇔ ⇓ (52% of pred. $\dot{V}$O_2peak_; < 40% pred. in 15%)	Deconditioning as major cause followed by cardiac involvement and lastly pulmonary limitations
Johnsen et al. [30]	*N* = 57 51 ± 13 y, 49% male 13 (22) days (spirometry and DLCO data are presented for full sample) ex-/smoker = 39%/2% diabetes = 9% CPET subsample: *N* = 31 NR, NR, NR	Mild (*n* = 23), moderate to critical (*n* = 34)—further categorisation not possible	Inclusion: patients evaluated in respiratory outpatient clinic 3 months after discharge (hospitalised group) or resolution of the acute disease for patients referred by their general physician. For CPET: symptomatic patients; abnormal lung function and/or if high-resolution computed tomography scans demonstrated significant pathology; able to perform CPET and understand study procedures Exclusion: NR	Assessed at 3 months post-discharge or following active COVID-19 for non-hospitalised individuals: $\dot{V}$O_2peak_ ? 27.5 ± 8.9 mL^.^min^−1.^kg^−1^ (two patients < 85% of pred.) Spirometry parameters ⇔ ⇓ DLCO ⇓ (74% of pred.) Breathing reserve ⇔ ⇓ (< 25% in 11 patients)	Decreased physical fitness (*N* = 11, 35.5%) or decreased ventilatory capacity (*N* = 5, 16%) as main reason for abnormal CPET
Acar et al. [44]	*N* = 51 42 (NR) y, 55% male NR ex-/smoker = NR diabetes = NR	Moderate (*n* = 34), severe (*n* = 17)	Inclusion: patients who survived the second wave of the COVID-19 pandemic Exclusion: contraindications to CPET (i.e. unstable angina, arrhythmia, severe aortic stenosis, heart failure, pulmonary hyper-tension, atrioventricular blocks, or severe hypertension)	Assessed at 3 months post-NR: $\dot{V}$O_2peak_ ⇓ (85% of pred.) Spirometry parameters ⇔ ⇓ (comparison with normative data NR) $\dot{V}$E/$\dot{V}$CO_2_ ⇔ (mean slope: 25) $\dot{V}$O_2_/WR slope ⇓ (5.6) O_2_ pulse ⇔ (Hb NR)	Decreased CRF due to peripheral muscle involvement
Jahn et al. [31]	*N* = 35 58 ± 13 y, 83% male 14 (15) days ex-/smoker = 29%/3% diabetes = 20%	Moderate (*n* = 18), severe (*n* = 14), critical (*n* = 3)	Inclusion: patients with RT-PCR-confirmed SARS-CoV-2 infection admitted to the hospital Exclusion: NR	Assessed at 3 months post-COVID-19 pneumonitis: $\dot{V}$O_2peak_ ⇓ (82% of pred.) Spirometry parameters ⇔ ⇓ DLCO ⇔ ⇓ (88% of pred.) Breathing reserve ⇔ O_2_ pulse ⇔ (Hb NR)	Deconditioning is the most common cause of impaired CRF in patients after severe COVID-19 pneumonitis
Joris et al. [16]	*N* = 14 59 (10) y, 71% male 40 (18) days ex-/smoker = NR/7% diabetes = 38%	Critical (*n* = 14)	Inclusion: surviving an intensive care unit stay > 6 days Exclusion: NR	Assessed at 3 months post-discharge: $\dot{V}$O_2peak_ ⇓ (81% of pred.) Spirometry parameters NR DLCO NA $\dot{V}$E/$\dot{V}$CO_2_ ⇔ (mean slope: 29) HR_peak_ ⇓ (71% of pred.) most patients received β-blocker O_2_ pulse ⇔ (Hb NR) VT1 ⇔ (78% of $\dot{V}$O_2peak_)	Decreased CRF mainly related to metabolic disorders rather than cardiac or pulmonary residual impairments
Rinaldo et al. [32]	*N* = 75 56 ± 13 y, 65% male NR ex-/smoker = 26%/12% diabetes = 12%	Mild-moderate (*n* = 18), severe (*n* = 18), critical (*n* = 39)	Inclusion: COVID-19 patients who recovered from the acute phase; age > 18 years; RT-PCR diagnosis of SARS-CoV-2 infection Exclusion: no informed consent; acute respiratory exacerbation in the 4 weeks before enrolment; contraindications for CPET (i.e. acute or unstable cardio-respiratory conditions, osteo-muscular impairment compromising exercise performance)	Assessed at 96 days post-discharge: $\dot{V}$O_2peak_ ⇓ (those below 85% of pred. [*n* = 41] had 83% of pred. and those above [*n* = 34] had 97% of pred.) Spirometry parameters: ⇔ DLCO ⇓ (normal CRF: 74% of pred.; reduced CRF: 69% of pred.) $\dot{V}$E/$\dot{V}$CO_2_ slope ⇔ (elevated in 15% of patients; reduced CRF: 29; normal CRF: 28) $\dot{V}$O_2_/WR slope ⇓ (reduced CRF: 9.9; normal CRF: 11.0) HRR ⇑ (but HR_peak_ ⇔) O_2_ pulse ⇓ (85% of pred.) (Hb NR) VT1 ⇔ ⇓ (reduced CRF: 48% of $\dot{V}$O_2max_ pred. [reduced in 37%]; normal CRF: 62% of $\dot{V}$O_2max_ pred.)	Reductions likely due to muscle deconditioning as a consequence of direct effect of viral load on muscle and/or physical inactivity. No relevant functional sequelae on ventilator and gas exchange response during exercise
Szekely et al. [4]	*N* = 71 53 ± 16 y, 66% male NR ex-/smoker = NR/11% diabetes = 13% Historical controls: *N* = 35, matched for age, sex, weight, height, hypertension and diabetes	Mild (*n* = 21), moderate (*n* = 37), severe (*n* = 10), critical (*n* = 3)	Inclusion: all patients with COVID-19 evaluated in the emergency department at the Tel Aviv Medical Center, ranging from mild to critical acute disease according to the National Institutes of Health definitions Exclusion: inability to provide informed consent and refusal to participate in the study	Assessed at 91 days post-symptom-onset: $\dot{V}$O_2peak_ ⇓ (29% lower than controls) Spirometry parameters ⇔ $\dot{V}$E/ $\dot{V}$CO_2_ at VT1 ⇔ HR_peak_ ⇓# Cardiac output ⇓ (stroke volume ⇓)# VT1 ⇔ ⇓ (# when expressed as rel. $\dot{V}$O_2_ but not in absolute terms) a-vO_2_ diff ⇑#	Cardiovascular mechanisms as main reason for low CRF. Chronotropic incompetence, reduced stroke volume and low peak a-vO_2_ diff contribute. Pulmonary limitations were rare
Mohr et al. [33]	*N* = 10 56 (12) y, 60% male 22 (19) days ex-/smoker = 10%/10% diabetes = 0%	Mild (*n* = 5), moderate (*n* = 2), severe (*n* = 3)	Inclusion: > 17 years of age; post-COVID-19 Still symptomatic with dyspnoea Exclusion: not fulfilling any of the above criteria; no CPET performed; any other reason for dyspnoea, underlying lung disease unrelated to COVID-19 judged responsible for patient’s dyspnoea	Assessed at 115 days post-discharge: $\dot{V}$O_2peak_ ⇓ (73% of pred.) Spirometry parameters ⇔ DLCO ⇓ 73% Breathing reserve ⇔ $\dot{V}$E/$\dot{V}$CO_2_ slope ⇔ (elevated in 20% of patients; mean slope: 31) HR_peak_ ⇓ (78% of pred.) O_2_ pulse ⇔ (Hb NR) VT1 ⇔ (73% of $\dot{V}$O_2peak_)	Muscular deficiency and thus metabolic limitations as main mechanism
Vonbank et al. [55]	*N* = 100 47 ± 13 y, 64% male NR ex-/smoker = 32%/9% diabetes = 13%	Asymptomatic (*n* = 3), mild (*n* = 76), moderate (*n* = 18), severe to critical (*n* = 3)	Inclusion: patients that recovered from asymptomatic or symptomatic COVID-19, confirmed by positive RT-PCR test Exclusion: NR	Assessed at 4 months post-diagnosis: $\dot{V}$O_2peak_ ⇔ ⇓# (Mild: 100% of pred.; moderate to critical: 86% of pred.; controls: 108% of pred.) Spirometry parameters ⇔ ⇓ # DLCO ⇔ ⇓ (Mild: 85% of pred.; moderate to critical #: 75% of pred.; controls: 83% of pred.) Breathing reserve ⇔ $\dot{V}$E/$\dot{V}$CO_2_ slope ⇔ (elevated in 20% of patients) HR_peak_ ⇓ # (only in moderate to critical but not mild) VT1 ⇔ ⇓ (Mild: 53% of $\dot{V}$O_2max_ pred.; moderate to critical #: 46% of $\dot{V}$O_2max_ pred.; controls: 55% of $\dot{V}$O_2max_ pred.)	Aside from impaired pulmonary function, cardiac and skeletal muscle dysfunction contributed to low CRF

COVID-19 severity was categorised according to the World Health Organisation interim guidance [12] whenever possible. Colour coding: Dots from left to right represent mild (not hospitalised), moderate, severe, and critical COVID-19, respectively. If a dot is coloured, this group is included in the particular study

*a-vO*_*2*_* diff* arteriovenous oxygen difference, *COVID-19* coronavirus disease 2019, *CPET* cardiopulmonary exercise testing, *CRF* cardiorespiratory fitness, *DLCO* lung diffusion capacity using carbon monoxide, *Hb* haemoglobin, *HR*_*max*_ maximal heart rate, *HR*_*peak*_ peak heart rate, *NR* not reported, *pred.* predicted, *SD* standard deviation, $\dot{V}$*E/*$\dot{V}$*CO*_*2*_ ventilatory efficiency, $\dot{V}$*O*_*2peak*_ peak oxygen uptake, *VT1* ventilatory threshold 1 (defined in [16]), *WR* work rate, # significantly different from control group; ⇑ increased; ⇓ decreased; ⇔ normal, ⇔ ⇑ slightly increased; ⇔ ⇓ slightly decreased

**Table 3:** Characteristics, reported outcomes, and author’s conclusions of studies examining the long-term sequelae of COVID-19 (sorted in descending order by time since hospital discharge/infection)

Study	Patients post-COVID-19 (*n*) Median (SD) age (y), male sex (%) Median (SD) hospital stay (d)	COVID-19 severity	Inclusion/exclusion criteria	Outcomes	Authors’ conclusion: reason for low CRF
Dorelli et al. [34]	*N* = 28 55 (10) y, 79% male 6 (7) days ex-/smoker = 32% diabetes = NR	Medical ward (*n* = 23), intensive care ward (*n* = 5) corresponds to moderate, severe and critical—further categorisation not possible	Inclusion: adults previously hospitalised for interstitial pneumonia due to COVID-19 Exclusion: age > 65 years; all concomitant previous respiratory or non-respiratory diseases, chronic respiratory failure or need for oxygen therapy under exertion; moderate obesity (BMI > 35 kg/m^2^); inability to perform functional tests; inability to perform CPET with a peak respiratory exchange ratio < 1.05; no chronic diseases, only stable arterial hypertension	Assessed at 169 days post-discharge: $\dot{V}$O_2peak_ ⇓ (if compared to [83]) Spirometry parameters ⇔ DLCO ⇔ ⇓ (90% of pred.) Breathing reserve ⇔ $\dot{V}$E/$\dot{V}$CO_2_ slope ⇔ (elevated in 29% of patients; absolute: 28) $\dot{V}$O_2_/WR slope ⇔ (11.8) VT1 ⇔ (60% of $\dot{V}$O_2peak_)	Normal lung function at rest. However, more than one-fourth of patients present with ventilatory inefficiency. This might be a sign of systemic alterations
Joris et al. [16]	*N* = 14 59 (10) y, 71% male 40 (18) days ex-/smoker = NR/7% diabetes = 38%	Critical (*n* = 14)	Inclusion: surviving an intensive care unit stay > 6 days Exclusion: NR	Assessed at 6 months post-discharge: $\dot{V}$O_2peak_ ⇓ (80% of pred.) Spirometry parameters ⇔ ⇓ DLCO ⇓ (71% of pred.) $\dot{V}$E/$\dot{V}$CO_2_ ⇔ (absolute: 28) HR_peak_ ⇓ (79% of pred.) O_2_ pulse ⇔ (Hb NR) VT1 ⇔ (72% of $\dot{V}$O_2peak_)	Low CRF mainly related to metabolic disorders rather than cardiac or pulmonary residual impairments
Cassar et al. [17]	*N* = 46 55 (13) y, 63% male 9 (12.5) days ex-/smoker = 37% diabetes = 17% Controls: *N* = 30, matched for age, sex, body mass index, risk factors, i.e. smoking, hypertension, diabetes, coronary artery disease, and stroke	Moderate (*n* = 29), severe (*n* = 17)	Inclusion: moderate to severe laboratory-confirmed COVID-19, admitted for inpatient treatment Exclusion: NR	Assessed at 3 months post-infection: See Raman et al. [42] Assessed at 6 months post-infection: $\dot{V}$O_2peak_ ⇔ ⇓# (93% of pred.; 31% below 80% of pred.) Spirometry parameters ⇔ (only FVC#) DLCO ⇔⇓ (81% of pred.) (impaired in 52%) $\dot{V}$E/$\dot{V}$CO_2_ slope ⇔ ⇑# (absolute: 31) O_2_ pulse ⇔ (Hb NR)# VT1 ⇓ (42% of $\dot{V}$O_2max_ pred.#; 30% below 40% of $\dot{V}$O_2max_ pred.)	Dissociation between persistent cardiopulmonary symptoms and CPET parameters Low CRF may persist due to symptomatic limitation and muscular fatigue. Reduced muscle mass and alterations in skeletal muscle metabolism are likely contributors
Vannini et al. [35]	*N* = 41 57 ± 14 y, 61% male NR ex-/smoker = NR/NR diabetes type 2 = 34%	Mild pneumonia (*n* = 12), severe pneumonia (*n* = 20), acute respiratory distress syndrome (*n* = 9) corresponds to moderate, severe, and critical—further categorisation not possible	Inclusion: consecutive patients dismissed after hospitalisation with a diagnosis of SARS-CoV-2 pneumonia; age > 18 and < 75 years Exclusion: NR	Assessed at 6 months post-?: $\dot{V}$O_2peak_ ⇓ (81% of pred.) (46% below 80% of pred.) Spirometry parameters ⇔ ⇓ DLCO ⇔ ⇓ (15% < 80% of pred.) Breathing reserve ⇔ $\dot{V}$E/$\dot{V}$CO_2_ slope ⇔ (absolute: 29) O_2_ pulse ⇔ (Hb ⇔) Cardiac output ⇔ ⇑	Deconditioning or circulatory causes cannot be asserted as the most common mechanism for low CRF
Debeaumont et al. [36]	*N* = 23 59 ± 13 y, 52% male 11 (10) days ex-/smoker = NR/NR diabetes = 17%	Moderate (*n* = 16), severe (*n* = 7)	Inclusion: patients referred to Rouen University Hospital for CPET due to persistent symptoms (fatigue or dyspnoea) following COVID-19-related hospitalisation Exclusion: history of chronic respiratory failure	Assessed at 6 months post-discharge: $\dot{V}$O_2peak_ ⇓ (84% of pred.) Spirometry parameters ⇔ DLCO ⇔ ⇓ (82% of pred.) Breathing reserve ⇔ $\dot{V}$E/$\dot{V}$CO_2_ slope ⇔ (absolute: 32) HR_peak_ ⇔ ⇓ (85% of pred.) O_2_ pulse ⇔ (Hb ⇔)	Persistent dyspnoea is likely caused by both persistent breathing disorder (overall high equivalents at VO_2_ peak and ventilatory inefficiency for those hospitalised in the ICU) and muscle deconditioning. No ventilatory limitation of CRF
Xiao et al. [37]	*N* = 56 48 ± 15 y, 50% male 18 ± 8 days ex-/smoker = NR/NR diabetes = 9% CPET subsample: *N* = 35 NR, NR NR NR NR	Moderate (*n* = 24), severe to critical (*n* = 11)	Inclusion: RT-PCR-confirmed SARS-CoV-2 infection accompanied by clinical manifestation and lung computer tomography changes, prior hospitalisation because of COVID-19 Exclusion: NR	Assessed at 6 months post-discharge $\dot{V}$O_2peak_ ⇓ (83% of patients < 80% of pred.) Spirometry parameters ⇔ ⇓ (FEV1/FVC: 9% of patients below 92% of pred.; FEV1: 17% of patients below 80% of pred.) $\dot{V}$E/$\dot{V}$CO_2_ slope ⇔ (22% of patients > 30) O_2_ pulse ⇓ (Hb NR [46% of patients < 80% of pred.]) VT1 ⇔ ⇓ abnormal in 20% defined as < 14 mL^.^min^−1.^kg^−1^)	COVID-19 may cause abnormal muscle metabolism as well as cardiopulmonary dysfunction (reduced O_2_-pulse in almost 46% and pulmonary dysfunction in 17% of patients)
Liu et al. [88]	*N* = 52 51 (16) y, 50% male 17 (11) days ex-/smoker = NR/NR diabetes = 2% CPET subsample: *N* = 37 NR, NR NR NR NR	Moderate (*n* = 24) and severe to critical (*n* = 13)	Exclusion: no computer tomography scan at admission or discharge, mild COVID-19 (i.e. no manifestation of pneumonia on chest computer tomography scan); history of lung cancer, tuberculosis or interstitial lung disease	Assessed at 6 months post-discharge: $\dot{V}$O_2peak_ ⇓ (abnormal in 54% of patients) Spirometry parameters: no comparison available $\dot{V}$E/$\dot{V}$CO_2_ slope ⇔ (absolute: 27.4) VT1 ⇔ ⇓ (abnormal in 19% defined as < 14 mL^.^min^−1.^kg^−1^)	No conclusions regarding possible underlying mechanisms
Aparisi et al. [38]	*N* = 70 55 ± 12 y, 36% male NR ex-/smoker = NR/NR diabetes = 6%	Mild to critical COVID-19—further categorisation not possible	Inclusion: prior hospitalisation because of COVID-19; patients not requiring hospital admission Exclusion: < 18 years of age; pregnancy; terminally ill; active SARS-CoV-2 infection; inability to exercise; previous known severe cardiopulmonary disease	Assessed at 181 days post-discharge: $\dot{V}$O_2peak_ ⇔ ⇓ (88% of pred.) Spirometry parameters ⇔ DLCO ⇔ ⇓ (89% of pred.) Breathing reserve ⇔ $\dot{V}$E/$\dot{V}$CO_2_ slope ⇔ (elevated in 29% of patients; absolute: 30) HR_peak_ ⇔ ⇓ (90% of pred.) O_2_ pulse ⇔ (Hb NR) VT1 ⇔ (79% of $\dot{V}$O_2peak_)	Perfusion / ventilation mismatch likely reflects gas exchange inefficiency or hyperventilation syndrome
Liu et al. [66]	*N* = 41 51 ± 14 y, 54% male 18 ± 7 days ex-/smoker = NR/NR diabetes = NR 29% of patients showed fibrosis	Moderate (*n* = 26), severe (*n* = 13), critical (*n* = 2)	Inclusion: prior hospitalisation because of COVID-19 Exclusion: NR	Assessed at 7 months post-discharge: $\dot{V}$O_2peak_ ⇓ # # (16.4 vs 20.2 mL^.^min^−1.^kg^−1^) Spirometry parameters ⇔ $\dot{V}$E/$\dot{V}$CO_2_ slope ⇔ # # (30.6 vs. 26.3) VT1: no comparison available	Chest computer tomography lesions could be absorbed without any sequelae for most patients, whereas older patients with severe conditions are more prone to develop fibrosis, which may further lead to cardiopulmonary insufficiency
Alba et al. [39]	*N* = 18 41 (23) y, 23% male NR ex-/smoker = 17%/0% diabetes type 2 = 6% Controls: *N* = 18, age- and sex-matched with unexplained dyspnoea and/or exercise intolerance unrelated to COVID-19	Mild (*n* = 12), moderate (*n* = 3), severe (*n* = 3) according to outpatient clinic (mild), medical ward (moderate) or ICU (severe)—further categorisation not possible	Inclusion: adult outpatients referred by the Massachusetts General Hospital Coronavirus Recovery Pulmonary Clinic for CPET between 01.08.2020 and 01.03.2021 with confirmed SARS-CoV-2 infection by RT-PCR test; chief complaint of persistent dyspnoea and/or exercise intolerance post-COVID-19 Exclusion: no confirmed SARS-CoV-2 infection by RT-PCR test; submaximal effort during CPET (respiratory exchange ratio < 1.0)	Assessed 258 days post-infection: $\dot{V}$O_2peak_ ⇓ (85% of pred.) DLCO ⇔ ⇓ (89% of pred.) Breathing reserve ⇔ $\dot{V}$E/$\dot{V}$CO_2_ slope ⇔ (absolute: 30) HR_peak_ ⇔ ⇓ (91% of pred.) O_2_ pulse ⇔ (Hb ⇔) VT1 ⇔ (135% of $\dot{V}$O_2max_ pred.)	Despite dyspnoea, only mild physiological abnormalities. Impaired DLCO was most prevalent functional finding
Mancini et al. [6]	*N* = 41 45 ± 13 y, 44% male 10 ± 7 days ex-/smoker = NR/NR diabetes = NR	Mild (*n* = 32), moderate to severe (n = 9)—further categorisation not possible	Inclusion: RT-PCR positive for SARS-CoV-2; developed new and persistent shortness of breath for > 3 months after recovery Exclusion: NR	Assessed on average 9 ± 3 months post-infection (range 3–15 months): $\dot{V}$O_2peak_ ⇓ (76% of pred.) Breathing reserve ⇔ VE/VCO_2_ slope ⇔ (absolute: 30.4) $\dot{V}$O_2_/WR slope ⇔ (9.7) HR_peak_ ⇓ (86% of pred.) O_2_ pulse (⇔?) (Hb NR) VT1 ⇔ (11.7 mL^.^min^−1.^kg^−1^ vs pred.: 10.6 mL^.^min^−1.^kg^−1^)	Most patients have circulatory impairment of CRF with dysfunctional breathing, suggesting reduced perfusion, especially pulmonary hypoperfusion
Singh et al. [58]	*N* = 10 48 ± 15 y, 10% male NR ex-/smoker = NR/NR diabetes = 0% Historical controls: *N* = 10, age- and sex-matched, exercise intolerance unrelated to COVID-19	Mild (*n* = 9), moderate (*n* = 1). Mild = not requiring hospitalisation, moderate = potentially requiring hospitalisation—further categorisation not possible	Inclusion: recovered from COVID-19, referred to hospital between February and June 2021 for unexplained exercise intolerance Exclusion: NR	Assessed at 11 months post-infection: $\dot{V}$O_2peak_ ⇓ # (70% of pred.) $\dot{V}$E/$\dot{V}$CO_2_ slope ⇑ # (absolute: 35; 1 of 10 patients < 30) HR_peak_ ⇔ ⇓ (85% of pred.) Cardiac output ⇔ a-vO_2_ diff ⇓ #	Peripheral limitation as major underlying mechanism. Systemic microcirculatory dysfunction and/or sarcopenia (described as myopathic process by authors)

COVID-19 severity was categorised according to the World Health Organisation interim guidance [12] whenever possible. *Colour coding*: Dots from left to right represent mild (not hospitalised), moderate, severe and critical COVID-19, respectively. If a dot is coloured, this group is included in the particular study

*a-vO*_*2*_* diff* arteriovenous oxygen difference, *COVID-19* coronavirus disease 2019, *CPET* cardiopulmonary exercise testing, *CRF* cardiorespiratory fitness, *DLCO* lung diffusion capacity using carbon monoxide, *FEV1* forced expiratory volume in 1 s, *FVC* forced vital capacity, *Hb* haemoglobin, *HR*_*peak*_ peak heart rate, *NR* not reported, *pred.* predicted, *SARS-CoV-2* severe acute respiratory syndrome-coronavirus-2, *SD* standard deviation, $\dot{V}$*E/*$\dot{V}$*CO*_*2*_ ventilatory efficiency, $\dot{V}$*O*_*2peak*_ peak oxygen uptake, *VT1* ventilatory threshold 1 (defined in [16]), *WR* work rate, # significantly different from control group; # # significantly different from group without fibrosis; ⇑ increased; ⇓ decreased; ⇔ normal, ⇔ ⇑ slightly increased; ⇔ ⇓ slightly decreased; ? unclear

In brief, the prevalence of restrictive lung disease seems to decrease with progressing recovery [4, 5, 17, 29–39]. The presented evidence indicates a negligible role of respiratory limitations detectable via spirometry to low $\dot{V}$O_2peak_, which is true for healthy individuals as well as patients recovering from COVID-19 (see Tables 1, 2, 3). Nonetheless, routine spirometry assessments may benefit patients with persistent restrictive lung disease and/or pre-existing respiratory disorders. Lung diffusion capacity measured by a single breath of carbon monoxide (DLCO) is of interest as a marker of diffusive limitations [17, 47]. DLCO may provide additional information on pulmonary-vascular limitations in the gear system [17, 27, 47]. As apparent from Fig. 2B, DLCO seems to recover slowly. Within the first 4 months post-infection, DLCO ranged between 69 and 84% of predicted [5, 16, 29, 32, 33, 42, 43]. Yet, even in the two studies showing higher mean values in DLCO (84% and 82%), 20% and 40% of patients were diagnosed with mild to moderate alterations [5, 43]. A longitudinal study examining patients 1.6 months and 6 months post-infection demonstrated that more than half of those patients without prolonged post-COVID-19 syndrome still had impaired DLCO [17]. Finally, at 6 and 9 months after hospital discharge, DLCO was around 90% of predicted [38, 39]. The combined evidence indicates that impairments in DLCO are more prevalent than restrictive lung disease in patients. This is supported by two meta-analyses in patients post-COVID-19 [28, 48], which showed that 6 months after discharge, 43% of patients still had a DLCO < 80% of predicted [48]. Considering this great fraction of patients with markedly low DLCO, impaired lung diffusion capacity may contribute to low $\dot{V}$O_2peak_ in some patients [49]. The association between $\dot{V}$O_2peak_ and DLCO is reported elsewhere [49].

As DLCO is a mathematical product of alveolar carbon monoxide uptake efficiency and alveolar volume, impairments may be due to either one or both of these parameters [50]. Limitations in alveolar carbon monoxide uptake efficiency may be explained by COVID-19-induced alveolar capillary damage, microvascular involvement or anaemia [51]. Restrictions in alveolar volume may be caused by microvascular injuries followed by the development of alveolar abnormalities with gradual loss of alveolar spaces [50]. Future studies might consider determining lung diffusion capacity by DLCO and additionally using nitric oxide (DLNO). DLNO has been suggested to be more sensitive than DLCO for detecting alterations in gas transport in patients post-COVID-19 [47, 52]. Gas transport abnormalities were proposed to be due to loss of alveolar units with alveolar membrane damage but relatively preserved capillary volume [47]. These may only be detected using nitric oxide [47]. Hyperpolarised xenon magnetic resonance imaging is a method that has lately been applied in some studies on COVID-19 [53]. Grist et al. [53] found impaired pulmonary gas transfer in seven of eleven non-hospitalised patients post-COVID-19 on average 287 days post-infection. The patients had normal CT scans [53]. These results and the strong correlation with DLCO findings suggest that hyperpolarised xenon magnetic resonance imaging may be a useful method to identify potentially undetected lung abnormalities in patients post-COVID-19 [53].

A normal to increased breathing reserve during exercise was commonly seen in studies with short-term [24, 40, 54], middle-term [4, 5, 16, 17, 29, 31–33, 43, 55], and long-term follow-up [6, 34–36, 38, 39] of patients post-COVID-19. This argues against respiratory limitations to V̇O_2peak_ but does not rule out impaired lung diffusion capacity.

The ventilatory equivalent (measured as $\dot{V}$E/$\dot{V}$CO_2_) is a widely studied parameter of CPET [56] and a marker for ventilatory efficiency. A $\dot{V}$E/$\dot{V}$CO_2_ > 35 is often considered an indicator of ventilatory inefficiency which is a hallmark of pulmonary arterial hypertension (although not specific to this condition) [56]. $\dot{V}$E/$\dot{V}$CO_2_ has thus been suggested as a valuable marker of early cardiovascular disease [56]. Notably, ventilatory inefficiency does not correspond well with reduced breathing reserve [57]. Ventilatory inefficiency may thus be present even with normal breathing reserve [57].

As apparent in Tables 1, 2, 3, the prevalence of ventilatory inefficiency up to 3 months post-hospital discharge ranged between 15 and 32% [32, 43], and remained around 10–29% thereafter [32–34, 38, 55, 58]. However, in 94% of studies, the mean of $\dot{V}$E/$\dot{V}$CO_2_ was < 35 (see Table 1 and Fig. 2C). We hypothesise that one of the mechanisms leading to ventilatory inefficiency may be induced by impaired pulmonary endothelial function and not respiratory limitations per se [56]. The thereby elicited changes such as pulmonary arterial obstruction, increased mean pulmonary arterial pressure, as well as a reduced capillary bed, may, in turn, lead to ventilation/perfusion mismatching [56]. This may ultimately result in dyspnoea and reduced $\dot{V}$O_2peak_ [56]. Consequently, examinations of ventilatory perfusion would provide valuable information regarding the involvement of the pulmonary vasculature.

Dysfunctional breathing is another aspect that should be discussed in light of the prevalence of ventilatory inefficiency as a potential contributing factor to exercise intolerance as well as post-COVID-19 symptoms such as dyspnoea or fatigue [42]. Skjørten et al. [5] found 3 months post-discharge that in those with ventilatory inefficiency (15% of the cohort), around 45% of cases could be attributed to dysfunctional breathing followed by circulatory (38%) and ventilatory limitations (17%). Motiejunaite et al. [43] likewise reported exercise hyperventilation as the main limitation in 16% of patients and a potential cause of dyspnoea. These studies show that dysfunctional breathing deserves investigation in patients post-COVID-19.

### Cardiovascular System (Gear 2)

The cardiovascular system is essential for the supply of oxygenated haemoglobin in the blood to the working muscles. Cardiovascular limitations can be identified via CPET by maximum heart rate being below the lower limit of normal, an abnormal increase of oxygen uptake or O_2_-pulse in relation to work rate, reduced ventilatory threshold 1 (VT1), or an abnormal heart rate/$\dot{V}$O_2_ slope (panels 2, 3, 4, 5 in the original Wasserman 9-panel graph) [27].

The supply capacity of O_2_ by the cardiovascular system is aside from the haemoglobin concentration in the blood reflected by cardiac output (stroke volume [SV] × heart rate) in Fick’s equation [9]. The majority of included studies reported O_2_-pulse as an estimate for SV calculated as oxygen uptake/heart rate at that stage [59]. Importantly, O_2_-pulse is equal to SV × arteriovenous oxygen difference [27]. It thus needs to be considered that in contrast to SV, O_2_-pulse may be altered by changes in the O_2_-carrying capacity of the blood, O_2_ uptake or heart rate when interpreting this parameter [59]. O_2_ uptake and heart rate can therefore directly impact the calculation of O_2_-pulse. A better capability of the mitochondria to utilise O_2_ would raise oxygen uptake and thereby lead to a higher O_2_-pulse, although SV may be indeed unaltered. Similarly, a lower maximum heart rate (e.g. as a consequence of COVID-19 or exercise training) would impact the calculation. It is thus not entirely clear whether the lower O_2_-pulse truly reflects a decreased SV or if it may be due to either chronotropic incompetence (failing to increase heart rate proportionately to work rate) [60], changes in the oxygen-carrying capacity and thus lower haemoglobin mass, or peripheral changes (i.e. arteriovenous oxygen difference) [59].

Up to 4 months post-infection, there were six studies [5, 16, 31, 33, 42, 44] reporting normal O_2_-pulse, of which three found at least mild chronotropic incompetence (see Tables 1, 2). Another study additionally performed stress-echocardiography, an indirect imaging method to obtain more precise estimates of cardiac output compared with O_2_-pulse [24]. Intriguingly, cardiac output did not differ between patients (*n* = 18) and controls despite lower O_2_-pulse and mild chronotropic incompetence in patients [24]. However, this was accompanied by a lower arterial blood O_2_-content than in controls [24]. While this demonstrates the difficulty of using O_2_-pulse as an estimate of SV, it shows that SV may indeed be (super-)normal since heart rate was below normal but cardiac output was similar to controls. The authors speculated that potentially persisting autonomic imbalance [24, 61] associated with suboptimal distribution of cardiac output to the working musculature could have led to low peripheral oxygen extraction [24]. This might, in turn, impair $\dot{V}$O_2peak_ [24]. These findings should, however, be interpreted with caution due to the small sample size. In contrast, lower estimated mean SV was seen in five studies between hospital discharge and 3.5 months post-infection by calculating O_2_-pulse [17, 24, 32, 43, 54]. While in Jahn et al. [31], O_2_-pulse was normal in the full sample, it was significantly lower when comparing patients stratified by low versus ‘normal’ $\dot{V}$O_2peak_ (defined as $\dot{V}$O_2peak_ > 82% of predicted). Moreover, one study assessed cardiac function by using stress-echocardiography 3 months after symptom onset [4]. Szekely et al. [4] observed lower left ventricular end-diastolic volume and ejection fraction, lower SV and lower cardiac output at all stages of exertion in patients compared with controls. In addition, the prevalence of chronotropic incompetence in this study was much higher in patients than in risk factor-matched controls (75% vs 8%) [4]. Following 4 months post-infection, SV and O_2_-pulse had normalised in most studies (see Table 2). The reversibility of low O_2_-pulse was further demonstrated by one longitudinal study [17], which showed that mean O_2_-pulse significantly improved from 91 to 95% of predicted up to 6 months post-infection. These findings are in line with a study by Vannini et al. [35] in patients with mild and severe COVID-19-induced pneumonia as well as acute respiratory distress syndrome, showing normal to supernormal cardiac output values at 6 months post-infection. Lastly, 11 months post-infection, cardiac output and peak oxygen delivery (calculated as arterial O_2_ content × cardiac output) were normal in a cohort with mainly mild COVID-19 but unexplained dyspnoea (*n* = 10) [58]. Apart from that, lower biventricular filling pressures, arguing against cardiovascular deconditioning, were apparent [58]. The authors concluded that cardiac limitations were absent and pointed toward the periphery as the main reason for impaired $\dot{V}$O_2peak_ [58]. Noticeable in this study was the pronounced reduction in $\dot{V}$O_2peak_ (70% of predicted) [58] compared with other studies ranging between 81 and 88% of predicted. However, small sample sizes and the choice of O_2_-pulse as an outcome measure might have been an issue in these studies. The Hamburg City Health Study (*n* = 443 patients post-COVID-19) found a slightly lower left-ventricular ejection fraction determined by echocardiography that was accompanied by higher levels of cardiac biomarkers in mostly non-hospitalised patients compared with controls at 10 months post-infection [45]. However, this difference was not present in cardiac MRI measures [45]. Interpretation of these results may therefore require some caution. In combination with low $\dot{V}$O_2peak_, another study reported 46% of patients being below 80% of predicted O_2_-pulse at 6 months post-discharge [37]. Because no heart rate data or information on arteriovenous oxygen difference were presented, it is unclear if the low O_2_-pulse was solely due to low SV [37]. Additionally to a low SV, a reduced maximum heart rate could impact the calculation of O_2_-pulse. These studies argue against SV-related long-term cardiac sequelae. Nonetheless, high-quality studies collecting data > 1 year post-infection using cardiac MRI or echocardiography may be valuable.

Importantly, not only SV but also maximum heart rate needs to be considered as a potential limitation of $\dot{V}$O_2peak_. At least mild chronotropic incompetence was present in ten studies (one study with two measurement points) with follow-ups exceeding 4 months [4, 6, 16, 32, 33, 36, 38, 39, 55, 58]. While chronotropic incompetence could directly impact O_2_ supply to the muscle, it is also an independent predictor of major cardiovascular events and mortality [60]. Interestingly, chronotropic incompetence is also associated with low $\dot{V}$O_2peak_ in patients with chronic fatigue syndrome [62]. These findings might thus have implications for patients post-COVID-19.

The $\dot{V}$O_2_/work rate slope is often abnormal in patients with cardiovascular diseases, thus reflecting limitations in the supply and/or metabolism of oxygen [27, 63]. Five studies reported $\dot{V}$O_2_/work rate slope [6, 32, 34, 44, 64]. Considering 10–11 mL min^−1^ W^−1^ as normal [63], results were inconsistent. In one study, $\dot{V}$O_2_/work rate slope was markedly reduced 3 months post-discharge (5.6 mL min^−1^ W^−1^, moderate and severe COVID-19) [44]. In the other studies, this parameter was only slightly below normal or normal (9.3, 9.9, 11.8 and 9.7 mL min^−1^ W^−1^ at 3 months to 9 months post-discharge) [6, 32, 34, 43]. Although most studies reported a $\dot{V}$O_2_/work rate slope that was slightly below the lower limit of the normal range, it was abnormal at least in a fraction of patients. This supports the findings of the above-presented parameters suggesting a potential contribution of cardiovascular factors to low $\dot{V}$O_2peak_. Again, the reader should be aware that $\dot{V}$O_2_/work rate slope may also be impacted by peripheral factors (discussed in Sect. 3.4) [27, 63].

VT1 is the point of exercise $\dot{V}$O_2_ at which the contribution of anaerobic pathways to energy production increases and, in turn, metabolic acidosis leads to a greater breathing stimulus [65]. VT1 is characterised by a change of slope of $\dot{V}$CO_2_ in relation to $\dot{V}$O_2_ or the inflection point of the $\dot{V}$E/$\dot{V}$O_2_ curve in relation to work rate [16]. It provides an indication of O_2_ supply limitations in the form of cardiac output, anaemia, pulmonary-vascular or peripheral-vascular factors, or a combination of those [65]. However, VT1 is not specific for deconditioning (see also Sect. 3.4) [65]. Eighteen studies presented data on VT1 reported as percentage of $\dot{V}$O_2peak_ or percentage of predicted $\dot{V}$O_2peak_ [4–6, 14–17, 29, 32–34, 37–39, 42, 54, 55, 66]. It needs to be emphasised that the unit reported has important implications for the interpretation of results. In a patient with a low $\dot{V}$O_2peak_, VT1 as percentage of $\dot{V}$O_2peak_ may be normal but VT1 as percentage of predicted $\dot{V}$O_2peak_ is reduced simply because of the lower measured $\dot{V}$O_2peak_. Alternatively, a reduced VT1 as percentage of $\dot{V}$O_2peak_ would always be accompanied by a reduced VT1 as percentage of predicted $\dot{V}$O_2peak_ independent of the level of $\dot{V}$O_2peak_. Most studies reported normal VT1 that was on average above 40% of $\dot{V}$O_2peak_. This also seemed to be the case for studies that reported VT1 as percentage of predicted $\dot{V}$O_2peak_ considering their actual $\dot{V}$O_2peak_ [4, 14–16, 29, 34, 38]. Yet, several studies reported abnormally low VT1 in 15–42% of patients up to 3 months post-infection [5, 32, 42] and in 19% and 20% of patients at 6 months post-discharge [37, 66]. This is again in line with the cardiovascular parameters discussed earlier, pointing towards a limitation in this organ system in a fraction of patients. The underlying mechanisms for these potential changes in cardiovascular parameters are still unclear. The harmful consequences of physical inactivity and especially bed rest during hospitalisation are extensively described in the literature [19, 20]. Cardiac performance reflected by SV and cardiac output has been shown to decline while heart rate increased, with the recovery of these parameters being delayed following bed rest [19]. Lower plasma volume, impaired vascular regulation, and cardiac atrophy may be responsible for these changes [19, 67]. Medications administered during the hospital stay such as steroidal treatment might lead to myopathic changes [23, 24]. Keeping the delayed recovery of cardiac output in mind, it seems suggestive that low $\dot{V}$O_2peak_, particularly in the first months post-infection, may at least partly be explained by deconditioning and treatment-related aspects affecting the cardiovascular system. The fact that at 4 months post-infection the SV is commonly reported to be close to normal (see Table 3) strongly suggests that the effect of deconditioning on $\dot{V}$O_2peak_ via reduced SV diminishes within a rather short time frame of 4 months. Because low $\dot{V}$O_2peak_ is prevalent up to 1 year post-infection, deconditioning alone is unlikely to be the only reason for low $\dot{V}$O_2peak_ after COVID-19. Nonetheless, it should be noted that deconditioning may also have peripheral manifestations [68].

Endothelial involvement caused by COVID-19-induced chronic inflammation could be another potential cause for the persisting limited cardiovascular function [21]. A recent review described the development of chronic inflammation months after acute COVID-19 infection that accompanies prolonged symptoms [21]. High levels of systemic inflammation, in turn, create a high risk for multi-organ damage [69, 70], potentially mediated by endothelial damage [71, 72]. It has been hypothesised that acute endothelialitis in the pulmonary circulation could extend into the systemic circulation, leading to myocardial injury as well as impaired function [72]. This impaired function might become apparent during CPET by reduced SV and/or chronotropic incompetence. Because none of the previous studies included an adequate measure of vascular function, such as flow-mediated dilation [73], this cannot be answered yet and warrants further investigation.

In conclusion, the contribution of the cardiovascular system to low $\dot{V}$O_2peak_ is unclear and the results are inconsistent. It seems that within the first few months post-infection a limited cardiac function contributes to low $\dot{V}$O_2peak_ in a considerable fraction of patients. In other patients, it appears that the main limitation lies in the periphery.

### Periphery/Musculature and Mitochondria (Gear 3)

Peripheral limitations are indicated by an abnormal response in respiratory exchange ratio, abnormal $\dot{V}$CO_2_ kinetics throughout exercise, a shallower increase of $\dot{V}$O_2_/work rate ratio, or reduced VT1 during CPET (panels 3, 5, 8 in the original Wasserman 9-panel graph) [27].

As discussed earlier on, $\dot{V}$O_2_/work rate ratio was abnormal at 3 months post-infection [44] and only slightly reduced or normal between 3 and 9 months post-infection (see Sect. 3.3) [6, 32, 34, 43]. VT1 was, on average, normal in most studies. However, as mentioned in Sect. 3.3, abnormal VT1 was documented in a fraction of patients up to 6 months post-infection [5, 32, 37, 42, 66]. Abnormal $\dot{V}$O_2_/work rate ratio and VT1 suggest a possible contribution of peripheral factors (i.e. peripheral-vascular or mitochondrial components) to low $\dot{V}$O_2peak_ in some patients. $\dot{V}$O_2_/work rate ratio and VT1, however, seem too unspecific to quantify the contribution of peripheral factors to low $\dot{V}$O_2peak_. It is also not possible to attribute abnormalities in these parameters to deconditioning or inflammation-induced changes that have distinct aetiologies, for example. However, several studies infer deconditioning due to abnormalities in $\dot{V}$O_2_/work rate ratio and VT1 [5, 31, 32]. Also, in some studies definitions for deconditioning seem to differ. Skjørten et al. [5] define deconditioning as the absence of ventilatory and cardiac exercise limitations, whereas in Rinaldo et al. [32] the definition also seems to include virus-induced alterations in muscle tissue. Consistent definitions thus need to be used in the future to obtain a better understanding of the underlying factors of low $\dot{V}$O_2peak_.

In the absence of O_2_ supply limitations, arteriovenous oxygen difference (a-vO_2_-diff) reflects the metabolic oxidative capacity, which is the ability of the mitochondria to utilise oxygen [9]. Measuring a-vO_2_-diff directly is complex [74] and thus is usually calculated using Fick’s equation, which was done in two of the three studies included in this review (both used stress echocardiography to determine cardiac output) [4, 24]. Because determining a-vO2-diff from solving Fick’s equation has severe limitations, such as errors introduced by indirect estimates of SV or the lag in $\dot{V}$O_2_ during incremental exercise [65], the results of these studies need to be interpreted with restraint. One study measured this outcome directly in patients with preserved cardiac output and in controls at 1 year post-infection. Patients showed a lower a-vO_2_-diff and peripheral oxygen extraction at peak exercise as compared with age- and sex-matched controls with exercise intolerance unrelated to COVID-19 [58]. These results indicate that peripheral alterations may contribute to low $\dot{V}$O_2peak_ even 1 year after discharge. In addition, Clavario et al. [29] evaluated maximal muscular strength using the dominant leg extension exercise. Based on the finding of muscular strength being independently associated with $\dot{V}$O_2peak_, the authors concluded that muscle impairment might be responsible for most of the functional diminishing [29]. However, as mentioned before, COVID-19-induced inflammation might also contribute to muscle loss [21]. Thus, it is questionable that the author’s data support the conclusion that deconditioning is the sole explanation for functional diminishing. Piotrowicz et al. [22] recently discussed the pathophysiology of post-COVID-19 acute sarcopenia. In their work, it becomes apparent that the structural and functional changes associated with sarcopenia also impact $\dot{V}$O_2peak_ [22]. This is in line with the proposed mechanisms by Singh et al. [58], likewise suggesting systemic microvascular dysfunction and a skeletal muscle myopathic process (i.e. sarcopenia) to be involved. To differentiate between inactivity- and COVID-19-related causes of muscle loss, biomarkers of inflammation and protein degradation may be helpful [75]. Muscle loss as a consequence of inactivity is commonly not accompanied by increased inflammation or muscle protein degradation as opposed to disease-induced changes [75]. For more details, the reader is referred to the review by Evans [75].

Based on the body of evidence, it is probable that both COVID-19-induced and inactivity-induced changes may lead to increased mitochondrial dysfunction, myofibrillar breakdown, reduced mitochondrial biogenesis as well as muscle synthesis and ultimately foster low $\dot{V}$O_2peak_ [22]. Given the parallels between patients with chronic fatigue syndrome and those with post-COVID-19, evidence on mitochondrial involvement in the former group of patients [76, 77] might be valuable. Although it is unlikely the primary cause of disease, mitochondrial dysfunction via immune-inflammatory and oxidative pathways is documented in chronic fatigue syndrome and closely related to exercise intolerance [76, 77]. These data may therefore support the conclusions regarding a peripheral contribution to low $\dot{V}$O_2peak_ in post-COVID-19. The effects of inactivity/bed rest on skeletal musculature are described elsewhere [68]. Considering that several studies suggested an underlying peripheral limitation, it seems to be important to bear these possible mechanisms of action in mind for further research as well as rehabilitation. Nonetheless, the extent of peripheral involvement in respect to the time post-infection, exact mechanisms and factors predisposing peripheral involvement are yet to be determined.

### Limitations

This review has some limitations. Raman et al. [78] highlighted that the heterogeneity in patient selection and factors such as virus variants, vaccines and choice of controls might have added to the variability in reported prevalence estimates. Indeed, infection with newly emerging SARS-CoV-2 variants such as Omicron might cause different post-COVID-19 sequelae and possibly a differential influence on $\dot{V}$O_2peak_ [79]. Also, within some of the studies included in this review, there was a wide heterogeneity in regard to the severity of COVID-19 in patients. Thus, the data shown in Fig. 2 could have led to an overestimation of the levels of $\dot{V}$O_2peak_ in severe COVID-19 and an underestimation in mild COVID-19 severity. COVID-19 vaccination status of patients might also affect $\dot{V}$O_2peak_. We previously demonstrated that physical activity, an important moderator of $\dot{V}$O_2peak_, differed between unvaccinated and fully vaccinated adults [80]. Finally, different methods to determine $\dot{V}$E/$\dot{V}$CO_2_ are available [56, 81]. However, the exact methods used were often not reported. Thus, we pooled $\dot{V}$E/$\dot{V}$CO_2_ data in Fig. 2C irrespective of the method used. This may have led to imprecision [81] but we believe this figure still provides a valuable overview of the data. The reader should be aware of these aspects.

Furthermore, there are limitations of the individual studies per se that need to be considered when interpreting the results. Firstly, the time at which the examinations were conducted was usually provided as either time since infection or since hospital discharge. This could have led to imprecision in our attempt to categorise studies into short, moderate, or long term as infection and hospital discharge may be separated by weeks or months. Secondly, all studies used an observational research design without pre-infection data. This makes it difficult to disentangle the contributions of pre-existing chronic conditions before the COVID-19 infection and lifestyle factors to low $\dot{V}$O_2peak_. Thirdly, as recently shown, individuals with low $\dot{V}$O_2peak_ seem to be more prone to greater COVID-19 severity than those with high $\dot{V}$O_2peak_ [7]. Simultaneously, the data from this review suggested a detrimental effect of COVID-19 on $\dot{V}$O_2peak_. Based on the available data, both are likely true, but it is unclear to which extent each factor contributes to low $\dot{V}$O_2peak_. Fourthly, most studies used normative data or historical controls for comparisons with cases. This introduces bias since the comparison was not subject to the potential influence of COVID-19 pandemic-related restrictions and their substantial impact on physical activity [82], a key determinant of $\dot{V}$O_2peak_ [19, 20]. Fifthly, the source of reference values remains unknown in nearly all studies, making it impossible for the reader to evaluate the generalisability of the results and compare the data across studies. Regarding $\dot{V}$O_2peak_, considerable differences between reference values have been shown recently [83]. Sixthly, most studies included hospitalised patients and/or those with persistent symptoms, limiting the generalisability to hospitalised patients with moderate to critical COVID-19. Seventhly, many studies focused exclusively on single gears, making it difficult to draw conclusions about potentially limiting factors on $\dot{V}$O_2peak_. Studies incorporating all parts of the gear system are thus needed to better understand the interplay of the three gears on low $\dot{V}$O_2peak_. To conclude, it is understandable that at an early stage of a pandemic, available clinical data are used to provide insights into potential sequelae quickly. However, to date, high-quality studies are missing.

## Conclusion

In patients post-COVID-19, as in healthy subjects, respiratory function seems to contribute in only a minor way to low $\dot{V}$O_2peak_. However, traditional spirometry parameters are measured at rest and may thus not reflect lung function during exercise. In contrast, the prevalence of low lung diffusion capacity, measured by DLCO, is markedly high in patients post-COVID-19, which might contribute to low $\dot{V}$O_2peak_ [49]. Future studies need to examine lung function during maximum performance and should include the combined measurement of DLNO-DLCO.

The cardiovascular system might contribute to low $\dot{V}$O_2peak_ via subnormal cardiac output due to chronotropic incompetence and lower SV, especially in the first months post-infection. At least mild chronotropic incompetence was similarly present at moderate- and long-term follow-up. Nonetheless, contrary findings exist. Larger cohort studies applying adequate methods to measure SV and consequently CO such as Doppler echocardiography or pulse contour devices are needed [84].

The fact that studies using CPET report the absence of major pulmonary or cardiac limitations suggests that the periphery, including the vasculature as well as musculature, might be central to impaired $\dot{V}$O_2peak_. To confirm this hypothesis, future studies using CPET need to determine muscle mass, muscle strength, muscle perfusion, mitochondrial function as well as a-vO_2_ diff.

The evidence presented in this review strongly suggests that it is not justified to declare deconditioning as the sole mechanism of low $\dot{V}$O_2peak_ as most studies have done (see Tables 1, 2 and 3). While deconditioning certainly explains part of the reductions, there are most likely other factors involved. A combination of COVID-19-induced and inactivity-induced processes might be responsible for the alterations in cardiac, vascular and muscular but also pulmonary function [21, 71, 72]. In addition, psychological factors may contribute substantially to the prolonged symptoms fostering exercise intolerance [85]. Schaeffer et al. [86], for instance, found lower $\dot{V}$O_2peak_ in patients with post-COVID-19 fatigue than in those without fatigue. This was accompanied by greater dyspnoea during exercise [86]. Psychological factors should thus not be disregarded in research, diagnosis or therapy/treatment.

The short- and long-term sequelae of COVID-19 are multifaceted and require diagnosis and treatment specific to the individual. This aligns well with recently proposed subtypes of post-COVID-19 syndrome that differ in terms of systematic manifestations as well as pathophysiological mechanisms [87]. Thorough testing with particular focus given to all three gear systems is required to receive a comprehensive understanding of the underlying causes of low $\dot{V}$O_2peak_ post-COVID-19. Such understanding would facilitate decision making in terms of diagnosis and treatment decisions. Thus, CPET is of paramount importance.

## Supplementary Information

Below is the link to the electronic supplementary material.

Supplementary file1 (DOCX 26 KB)
