# Supplementary material for: Low Cardiorespiratory Fitness Post-COVID-19: A Narrative Review
Source: Sports Med. 2022 Sep 17;53(1):51–74. doi: 10.1007/s40279-022-01751-7 (PMC9483283; doi:10.1007/s40279-022-01751-7)
Supplement: Supplementary file 1 — Supplementary file1 (DOCX 26 KB) [file 40279_2022_1751_MOESM1_ESM.docx]

**Low cardiorespiratory fitness post-COVID-19: a narrative review**

Schwendinger Fabian^1^, Knaier Raphael ^2,3^, Radtke Thomas^4^, & Schmidt-Trucksäss Arno^1^

^1^Division of Sports and Exercise Medicine, Department of Sport, Exercise and Health, University of Basel, Basel, Switzerland

^2^Division of Sleep Medicine, Harvard Medical School, Boston, MA, USA

^3^Medical Chronobiology Program, Division of Sleep and Circadian Disorders, Departments of Medicine and Neurology, Brigham and Women’s Hospital, Boston, MA, USA

^4^Epidemiology, Biostatistics and Prevention Institute (EBPI), University of Zurich, Zurich, Switzerland

Corresponding author: Arno Schmidt-Trucksäss (AST), arno.schmidt-trucksaess@unibas.ch; Department of Sport, Exercise and Health, University of Basel, Grosse Allee 6, 4052 Basel, Switzerland

Supplementary Information

**Online Resource 1.** Search strategy.

| **Database** | **Search strategy** |
| --- | --- |
| MEDLINE and  EMBASE on Ovid | ((((covid-19 or SARS-CoV-2).sh. or (coronavirus/ or betacoronavirus/ or coronavirus infections/)) and (disease outbreaks/ or epidemics/ or pandemics/)) or (nCoV* or 2019nCoV or 19nCoV or COVID19* or COVID or SARS-COV-2 or SARSCOV-2 or SARSCOV2 or Severe Acute Respiratory Syndrome Coronavirus 2 or Severe Acute Respiratory Syndrome Corona Virus 2).ti,ab,kf,nm,ot,ox,rx,px.) and ((((cardiorespiratory fitness or exercise test).sh. or (exercise capacity or cardiorespiratory fitness or oxygen uptake or aerobic capacity or endurance capacity or cardiopulmonary exercise test* or maximum oxygen uptake or peak oxygen uptake or maximal oxygen uptake or CPET or spiroergometry).ti,ab,kf.) and (follow-up studies.sh. or (long-term consequences or sequelae or follow-up* or post-infection or post-discharge or consequenc* or short-term or moderate-term or middle-term).ti,ab,kf.)) not (exp animals/ not humans/)) |
| L^.^OVE platform by Epistemonikos Search String (on L^.^OVE  Coronavirus disease [COVID-19] “advanced search beta”) | (exercise capacity OR cardiorespiratory fitness OR oxygen uptake OR aerobic capacity OR endurance capacity OR cardiopulmonary exercise test* OR maximum oxygen uptake OR peak oxygen uptake OR maximal oxygen uptake OR CPET or spiroergometry) AND (long-term consequences OR sequelae OR follow-up* OR post-infection OR post-discharge OR consequenc* OR short-term OR moderate-term OR middle-term)  Applied to full search: limit publication year to ”2019 -Current” |
